# Disease-modifying therapeutic directions for Lewy-Body dementias

**DOI:** 10.3389/fnins.2015.00293

**Published:** 2015-08-20

**Authors:** Qiang Zhang, Young-Cho Kim, Nandakumar S. Narayanan

**Affiliations:** ^1^Department of Neurology, University of IowaIowa City, IA, USA; ^2^Physician Scientist Training Program, University of IowaIowa City, IA, USA; ^3^Aging Mind and Brain Initiative, Carver College of Medicine, University of IowaIowa City, IA, USA

**Keywords:** Dementia with Lewy Bodies, Parkinson's disease, Alzheimer's disease, Deep Brain Stimulation, disease-modifying therapy

## Abstract

Dementia with Lewy bodies (DLB) is the second leading cause of dementia following Alzheimer's disease (AD) and accounts for up to 25% of all dementia. DLB is distinct from AD in that it involves extensive neuropsychiatric symptoms as well as motor symptoms, leads to enormous societal costs in terms of direct medical care and is associated with high financial and caregiver costs. Although, there are no disease-modifying therapies for DLB, we review several new therapeutic directions in treating DLB. We discuss progress in strategies to decrease the level of alpha-synuclein, to prevent the cell to cell transmission of misfolded alpha-synuclein, and the potential of brain stimulation in DLB.

## Introduction

Dementia with Lewy bodies (DLB) is the second most common pathologic diagnosis of dementia, following Alzheimer's disease (AD), comprising 25% of all dementias (Heidebrink, 2002; Mayo and Bordelon, 2014). Yet, DLB is underdiagnosed (Galvin and Balasubramaniam, 2013). The pathologic feature of DLB is the presence of Lewy bodies in the cortex and brainstem. Lewy-bodies are neuronal inclusions of abnormal filamentous assemblies of α-synuclein and ubiquitin (Spillantini et al., 1998). DLB is associated with high burdens of neurofibrillary tangles (Merdes et al., 2003) but lower levels of amyloid-β (Masliah et al., 2001).

DLB is characterized by fluctuating cognition and alertness, visual hallucinations, parkinsonian movement syndrome, REM sleep disorders, neuroleptic sensitivity, and reduced striatal dopamine. Due to the neuropsychiatric features of DLB, the costs for care of DLB patients is more than 2 times higher than AD patients (Bostrom et al., 2007; Ricci et al., 2009).

It is very difficult to distinguish DLB from dementia-associated with Parkinson's disease (PDD), which shares many underlying clinical and pathological features with DLB (Narayanan et al., 2013; Parker et al., 2013). Current guidelines arbitrarily distinguish DLB and PDD by the timing of onset of dementia in relation to motor symptoms (Lippa et al., 2007). One influential hypothesis is that synuclein spreads rostrally from the periphery (Braak et al., 2003). However, this hypothesis does not account for widespread cortical synuclein observed at initial diagnosis DLB.

## Targeting α-synuclein

The major component of Lewy bodies in DLB and Parkinson's disease (PD) is misfolded α-synuclein (Spillantini et al., 1998). The normal α-synuclein is a soluble protein and is involved in presynaptic processing of neurotransmitters, mitochondrial function and proteasome processing (Cheng et al., 2011). In DLB and PD, α-synuclein aggregates in Lewy bodies and causes neuronal death. The α*-synuclein* gene triplication and missense mutations, E46K and A53T, are associated with familial PD/PDD/DLB. Overexpression of human wild-type α-synuclein in mice leads to early cholinergic deficits and cognitive abnormalities, which appears before the development of motor deficits (Magen et al., 2012). Importantly, α*-synuclein* knockout mice have normal neuroanatomy (Abeliovich et al., 2000), normal movement (Kokhan et al., 2012) and normal learning behavior (Chen et al., 2002). Therefore, various strategies have been employed to reduce α-synuclein directly for the treatment of DLB and PD.

Nilotinib is an Abl tyrosine kinase inhibitor approved by FDA for the treatment of chronic myelogenous leukemia. Nilotinib decreased the level of α-synuclein and reverse the loss of dopamine neurons in a mouse model overexpressing A35T mutant α-synuclein (Hebron et al., 2013). It was also shown that the Abl inhibition through nilotinib promotes autophagic degradation of α-synuclein. Another study has shown that α-synuclein is a substrate of Abl and Abl directed phosphorylation leads to decreased α-synuclein degradation through the autophagy and proteasome pathways (Mahul-Mellier et al., 2014). A Phase I clinical trial of nilotinib is currently ongoing (http://clinicaltrials.gov/).

Secreted, extracellular α-synuclein might play a crucial role in the passage of misfolded α-synuclein from one cell to another (Lee et al., 2014). Therefore, immunotherapy targeting extracellular α-synuclein has been proposed (Masliah et al., 2005, 2011; Valera and Masliah, 2013). Masliah et al. (2011) found that immunization with recombinant human α-synuclein led to a reduction in α-synuclein accumulation and neurodegeneration without neuroinflammation. With promising results from active immunization, they then applied passive immunization to the same mouse model using 9E4, an antibody targeting the C terminal epitopes of α-synuclein. They found that 9E4 reduced the accumulation of α-synuclein aggregates in neocortex and hippocampus. They also found that 9E4 treatment ameliorated motor behavior and learning deficits, and improved synaptic pathology. Bae et al. (2012) found that administration of anti-α-synuclein antibody into the brains of PGDF-α-synuclein transgenic mice prevented cell-to-cell transmission of α-synuclein. The antibodies aid in clearance of extracellular α-synuclein proteins by microglia, thereby preventing their actions on neighboring cells. Misfolded extracellular α-synuclein might interact with antibodies to form antigen-antibody complexes, and these complexes are endocytosed and transferred to the lysosomal compartment for degradation through autophagy (Masliah et al., 2011). Antibody bound extracellular α-synuclein aggregates are also cleared by microglia cells (Bae et al., 2012). Tran et al. (2014) employed an antibody specific for misfolded α-synuclein and obtained promising results in animal models as well. Recently, AFFiRiS AG, an Austria-based biotech company, developed a vaccine targeting PD and other synucleinopathies. The peptides used in the vaccine are designed to be too small to induce an α-synuclein-specific T cell response, thus avoiding T cell autoimmunity (Mandler et al., 2014). The vaccine was tested in the PGDF-α-synuclein and the Thy1-α-synuclein transgenic mouse models. Active vaccination resulted in decreased accumulation of α-synuclein oligomers in axons and synapses, reduced neurodegeneration, and improvements in motor and memory deficits in both models. Phase I clinical trials are currently ongoing in early PD and multiple-system atrophy patients with PD01A and PD03A vaccines (http://clinicaltrials.gov/).

Another strategy targeting α-synuclein is RNA interference (RNAi) (Fire et al., 1998). Direct infusion of siRNA led to the reduction of α-synuclein (Lewis et al., 2008; McCormack et al., 2010). Recent studies have employed virally-mediated RNAi delivery. Sapru et al. (2006) used lentivirus-mediated RNAi to successfully silence human α-synuclein expression in the rat substantia nigra. Khodr et al. employed AAV-mediated RNAi, but found that this approach caused neurotoxicity (Han et al., 2011; Khodr et al., 2011, 2014). They then tried AAV-mediated RNAi embedded in mircoRNA30 backbone, and they were able to reverse α-synuclein induced forelimb deficit and dopaminergic neuron loss. However, this approach induced inflammation. Transgene delivery using AAV was shown to be safe in previous studies and this technology has been used in human clinical trials in PD (LeWitt et al., 2011).

Other approaches employed to reduce α-synuclein include ribozymes (Hayashita-Kinoh et al., 2006), intracellular expression of single chain antibodies (Zhou et al., 2004; Lynch et al., 2008; Yuan and Sierks, 2009), endogenous microRNA (Junn et al., 2009), and mirtrons (Sibley et al., 2012). A safe and effective approach to reduce the level of α-synuclein will likely slow down or even reverse the progression of DLB.

## Targeting synucleinopathy progression

α-synuclein spreads via prion-like mechanisms (Angot et al., 2010). Initial evidence came from postmortem PD brains who received transplants of fetal mesencephalic neurons over 10 years before death. Two studies independently found Lewy bodies in grafted neurons (Kordower et al., 2008; Li et al., 2008). Further, studies in animal models have confirmed the prion-like spreading synucleinopathy (Desplats et al., 2009; Hansen et al., 2011; Kordower et al., 2011; Volpicelli-Daley et al., 2011; Angot et al., 2012; Luk et al., 2012a,b; Mougenot et al., 2012; Masuda-Suzukake et al., 2013; Rey et al., 2013; Sacino et al., 2013; Reyes et al., 2014). Interestingly, recent studies have shown that brain extracts from multiple-system atrophy and PD patients triggered α-synuclein pathology in animal models (Watts et al., 2013; Recasens et al., 2014), in a way similar to the transmission of kuru disease to chimpanzees.

**Figure 1:**
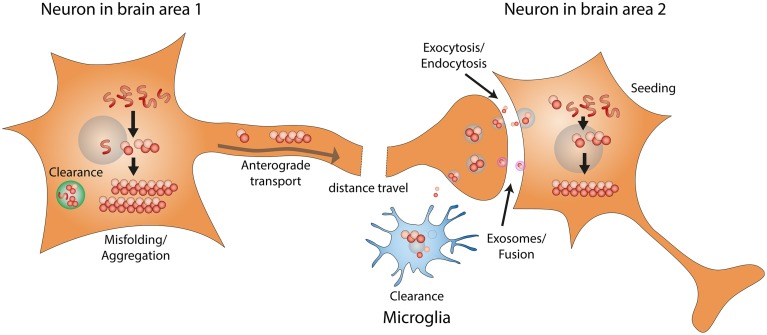
**Prion-like progression of synucleinopathy**. Endogenous α-synuclein misfolds and forms amyloid fibrils. α-synuclein amyloid fibrils that fail to be cleared act as seeds and convert more endogenous α-synuclein into misfolded amyloid fibrils. Through intracellular axonal transport, misfolded α-synuclein fibrils undergo long distance travel within the nervous system. Misfolded α-synuclein fibrils might be released by exocytosis or in exosomes. Misfolded α-synuclein can then be taken up by surrounding neurons, through endocytosis or direct fusion with exosomes. Misfolded α-synuclein fibrils taken up by surrounding neurons can recruit endogenous α-synuclein to form more amyloid fibrils, through the seeding mechanism.

As shown in Figure 1, the development/progression of α-synuclein pathology consists of the following steps: (1) misfolding/aggregation of α-synuclein, (2) induction of endogenous α-synuclein to form more aggregates by the misfolded α-synuclein aggregates (also known as “seeding”), (3) long distance transport of α-synuclein aggregates, (4) secretion/exocytosis of α-synuclein aggregates, (5) uptake/endocytosis of α-synuclein aggregates by surrounding neurons, (6) formation of more α-synuclein aggregates through “seeding” in the healthy neuron. Extensive studies have been performed to determine the details of each step, and potential therapeutic approaches are being developed to target the prion-like progression of synucleinopathy.

*In vitro* studies have shown that under certain conditions, recombinant α-synuclein forms amyloid fibrils spontaneously (Narkiewicz et al., 2014). Addition of small amount (0.001%) of preformed α-synuclein fibrils to unfolded recombinant α-synuclein solution could accelerate the formation of more fibrils, and this phenomenon is known as seeding (Wood et al., 1999). Strikingly, intrastriatal inoculation of recombinant α-synuclein fibrils in wild type mice led to Lewy pathology in anatomically interconnected regions (Luk et al., 2012a). Agents preventing the formation of α-synuclein fibrils or interfering with the seeding process could potentially serve as disease modifying treatments for both DLB and PDD. Efficient *in vitro* screening approaches and chronically α-synuclein “infected” cell lines have been established (Herva et al., 2014; Narkiewicz et al., 2014). These should allow for high-throughput screening of compounds targeting synucleinopathy (Rochet, 2007). Several agents targeting α-synuclein aggregation have been tested in cellular or animal models with promising results (El-Agnaf et al., 2004; Jiang et al., 2010; Zhou et al., 2011; Wagner et al., 2013). On the other hand, avoiding environmental exposure to agents associated with induction of synucleinopathy is very important (Elbaz et al., 2009).

It is well established that α-synuclein is involved in vesicular trafficking and release (Burre et al., 2010; Nemani et al., 2010), and that it is transported along the axons (Jensen et al., 1999; George et al., 2013). Importantly, recent cell culture studies have utilized microfluidic chambers to separate neuronal cell bodies from their terminals, and demonstrated that α-synuclein fibrils can be transported along the axons in both directions (Danzer et al., 2011; Volpicelli-Daley et al., 2011; Freundt et al., 2012). Freundt et al. (2012) used live-cell imaging and immunofluorescence, and confirmed the axonal transport of fluorescent α-synuclein fibrils, and their transfer to second-order neurons through axon-to-soma transfer. In order to confirm the long distance transport of misfolded α-synuclein in a synucleinopathy mouse model induced by intragastric administration of rotenone, Pan-Montojo et al. (2012) performed resection of the autonomic nerves and found that the progression of synucleinopathy was blocked. These results strongly indicate that the progression of synucleinopathy is based on the transneuronal and axonal transport of misfolded α-synuclein.

A prerequisite of cell to cell transmission is the secretion and uptake of α-synuclein fibrils. α-synuclein is present in human plasma, cerebrospinal fluid, and brain interstitial fluid (Borghi et al., 2000; El-Agnaf et al., 2003; Emmanouilidou et al., 2011). α-synuclein that is in the CSF and brain interstitial fluid is derived from CNS neurons (Mollenhauer et al., 2012), is present in cytoplasmic vesicles and could be secreted through exocytosis (Lee et al., 2005; Jang et al., 2010). Exocytosis of α-synuclein and translocation of α-synuclein into vesicles increases under various stress conditions (Jang et al., 2010). Intravesicular α-synuclein was found to be more prone to aggregation and aggregated forms of α-synuclein are also secreted from cells (Lee et al., 2005). Exocytosis of α-synuclein was found to be independent of the ER-Golgi classical pathway, and exosome-associated exocytosis has been implicated in the release of misfolded α-synuclein and the cell-to-cell transmission of synucleinopathy (Lee et al., 2005; Emmanouilidou et al., 2010). Exosome-associated exocytosis was thought to be directed by the multivesicular bodies–intraluminal vesicles mediated pathway (Emmanouilidou et al., 2010). In addition to exocytosis, the multivesicular bodies–intraluminal vesicles pathway also goes toward lysosomal degradation. It has been found that lysosomal dysfunction increases exosome-associated α-synuclein release and transmission (Alvarez-Erviti et al., 2011). Interestingly, Hasegawa et al. (2011) found that impaired biogenesis of multivesicular bodies was associated with hyper-secretion of α-synuclein. α-synuclein could also be released through the Rab11a-dependent recycling endosome pathway, and the blockage of multivesicular bodies mediated lysosomal degradation of α-synuclein could have contributed to the hyper-secretion of α-synuclein (Liu et al., 2009). Once released to the extracellular space, free misfolded α-synuclein could get internalized by nearby neurons through endocytosis, and exosome-associated α-synuclein could enter cytosol directly by fusion of the exosome with the plasma membrane (Desplats et al., 2009; Danzer et al., 2012). Immunotherapy approaches targeting extracellular α-synuclein were discussed in the previous section. Strategies targeting the exocytosis and endocytosis processes are being considered, but these should be approached with caution, as severe side effects might ensue (Lee et al., 2014). In the meanwhile, other mechanisms have also been proposed for the pathogenesis of DLB: mitochondrial dysfunction, abnormal calcium handling, and altered inflammatory responses (Goedert et al., 2013; Mullin and Schapira, 2013; Overk and Masliah, 2014; Zaltieri et al., 2015). Also, it is important to note that some studies have provided evidences that argue against the concept of prion-like progression in synucleinopathy (Hallett et al., 2014).

## Deep brain stimulation

In addition to α-synuclein centered approaches, very recently, much attention has been attracted to another highly promising approach: Deep Brain Stimulation. Deep brain stimulation (DBS) has been approved by the FDA for the treatment of PD (Deuschl et al., 2006; Follett et al., 2010), essential tremor, dystonia, and obsessive-compulsive disorder (Rezai et al., 2008). It has been employed in research studies to treat chronic pain and major depressive disorder (Plow et al., 2012; Blumberger et al., 2013). Recently, DBS has been proposed in the treatment of cognitive disorders like AD (Sankar et al., 2014). Currently, two Phase I clinical studies are being conducted for the treatment of DLB with DBS targeting the nucleus basalis of Meynert (NBM) (http://clinicaltrials.gov/). Here, we review DBS approaches that are directly and indirectly related to treating DLB.

**Figure 2:**
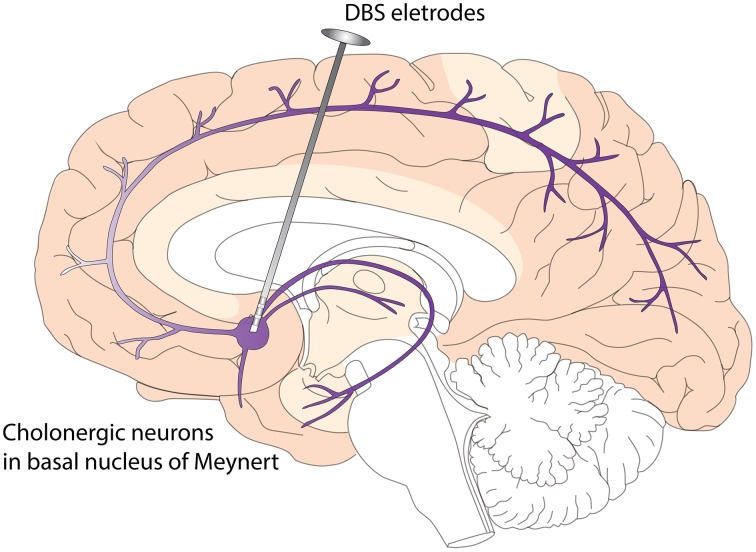
**Deep Brain Stimulation (DBS) of Nucleus Basalis of Meynert (NBM) in Dementia with Lewy Bodies (DLB)**. NBM is one group of the basal forebrain cholinergic neurons (BFCN). The degeneration of BFCN is associated with decreased cholinergic tone and cognitive deficits in DLB. NBM provides cholinergic innervations to the entire neocortex. DBS of NBM likely enhances the cholinergic tone of neocortex, leading to improved cognitive function and quality of life. Potential disease modifying mechanisms include enhanced neurogenesis and increased nerve growth factor trophic support.

DBS has the potential to modulate cognition. In PDD patient, Freund et al. (2009) and Barnikol et al. (2010) performed DBS targeting the NBM, a cholinergic nucleus. They found markedly improved cognition. This is the first and only published study targeting synucleinopathy associated dementia with DBS. This PDD patient received bilateral implantation of DBS leads into the STN and NBM. While isolated STN stimulation led to motor improvement without significant change in cognitive function, the addition of NBM stimulation is associated with improvement in cognitive functions. NBM stimulation also led to significant improvement in personality features, social communication, and overall quality of life. The authors believe that these effects were likely related to the effects of stimulating residual cholinergic projections and cell bodies in NBM. At this point, it is not clear whether DBS in NBM influences the progression of PDD or DLB, but two disease modifying mechanisms have been proposed based on preclinical studies. These include increased nerve growth factor (NGF) secretion and enhanced neurogenesis (Hotta et al., 2009; Jeong Da et al., 2014) (Figure 2). In terms of short term effects of NBM DBS, animal studies have indicated that NBM DBS is linked to brain plasticity and cortical map reorganization(Kilgard and Merzenich, 1998), and associated with enhanced learning and induction of immediate-early gene c-Fos (Boix-Trelis et al., 2006, 2009).

Basal forebrain cholinergic neurons (BFCN), consisting of NBM, medial septum and diagonal band of Broca, provide cholinergic innervations to the neocortex, entorhinal cortex and hippocampus. It has been found that NGF is very important for the survival and function of BFCN, and it has been shown that the majority of NGF nurturing BFCN comes from targeting areas of BFCN (neocortex, entorhinal cortex, and hippocampus) and is transported retrogradely through the axons back to the cell bodies of BFCN (Isacson et al., 2002). Interestingly, NBM DBS led to increased NGF secretion in wild type rats (Hotta et al., 2007, 2009), indicating that DBS could potentially reverse BFCN degeneration through increasing NGF trophic support. In another study, Jeong Da et al. (2014) injected 192 IgG-saporins intraventricularly to induce cholinergic lesion in rats. They found that DBS in the medial septum restores spatial memory in the setting of cholinergic neuron damage, and that this effect is associated with an increase in hippocampal cholinergic activity and neurogenesis.

NBM DBS was first applied to an AD patient three decades ago (Turnbull et al., 1985). Turnbull et al. applied cyclic DBS to the left NBM for 9 months, and glucose utilization was relatively preserved in the left cerebral hemisphere of this AD patient. However, DBS did not lead to clinical improvement on memory or other cognitive measures. Recently, Kuhn et al. (2015) performed a Phase I clinical trial of NBM DBS on 6 mild to moderate AD patients. No safety concern was noted, and promising positive effects were observed on cognitive parameters including Alzheimer's Disease Assessment Scale-Cognitive subscale and Mini Mental Status Exam, global functioning as measured by Clinical Dementia Rating, subjectively perceived quality of life, and increased cortical glucose metabolism (via PET).

DBS targeting fornix, part of the Papez circuit which is very important in memory retention, has also been tested in AD patients, with promising results (Laxton et al., 2010). A Phase II clinical trial of Fornix DBS is currently underway in AD patients. Studies on human subjects have also indicated that DBS of entorhinal cortex (Suthana et al., 2012), anterior thalamic nucleus (Oh et al., 2012), pedunculopontine tegmental nucleus (Stefani et al., 2010), rhinal cortex and hippocampus (Fell et al., 2013) might also enhance memory and cognitive functions. Animal studies with DBS targeting fornix (Hescham et al., 2013), entorhinal cortex (Stone et al., 2011), anterior thalamic nucleus (Hamani et al., 2011; Chen et al., 2014) and hippocampus (Berger et al., 2011) have also indicated positive effects on memory and cognitive functions.

Animal studies have targeted other areas of the brain and evaluated the effects of DBS on cognitive functions (Hardenacke et al., 2013). Promising results were obtained through DBS targeting anterior caudate nucleus (Williams and Eskandar, 2006), midline thalamic nuclei (Arrieta-Cruz et al., 2010), central thalamus (Shirvalkar et al., 2006; Mair and Hembrook, 2008; Shah et al., 2009; Smith et al., 2009), lateral hypothalamus (Soriano-Mas et al., 2005, 2007; Ruiz-Medina et al., 2008a,b; Huguet et al., 2009; Aldavert-Vera et al., 2013), and amygdala (Kadar et al., 2011).

Given its low complication rate and reversibility (Deuschl et al., 2006; Voges et al., 2006), DBS seems to be a safe and promising approach in DLB management. Though long term disease modifying profile is not clear at this point, DBS will likely improve short term cognitive functions, and importantly, the quality of life of patients with DLB and PDD (Freund et al., 2009). Though DBS targeting fornix has attracted much attention in the field of AD treatment, NBM DBS currently holds the most promise for DLB patients. Unlike AD, which usually presents with memory deficits, DLB usually presents with fluctuating cognition and visuospatial deficits with relative sparing of memory in earlier stages (Mayo and Bordelon, 2014). While waiting for the results of ongoing clinical trials, it is worth noting that no DBS studies have been performed on animal models of dementia associated with synucleinopathy. These preclinical studies will likely accelerate and validate clinical application of DBS in DLB and other synucleinopathy associated dementia.

## Conclusion

DLB is the second leading cause of dementia following AD, and disease modifying therapies are urgently needed. Significant progress has been made in the past decade regarding the underlying mechanism of DLB pathogenesis. Future therapies might modify the progression of DLB by targeting α-synuclein or by stimulating deep brain structures. Cutting edge research into the basic mechanism of DLB pathogenesis and preclinical studies testing novel therapies will continue to play crucial roles in the development of disease modifying treatments for DLB.

### Conflict of interest statement

The authors declare that the research was conducted in the absence of any commercial or financial relationships that could be construed as a potential conflict of interest.

## Abbreviations

AD: Alzheimer's disease
BFCN: Basal forebrain cholinergic neurons
DBS: Deep brain stimulation
DLB: Dementia with Lewy bodies
NBM: nucleus basalis of Meynert
PDD: dementia-associated with Parkinson's disease
PD: Parkinson's disease
RNAi: RNA interference.
